# Pyrrolobenzodiazepine Antibody-Drug Conjugates Designed for Stable Thiol Conjugation

**DOI:** 10.3390/antib6040020

**Published:** 2017-11-28

**Authors:** R. James Christie, Arnaud C. Tiberghien, Qun Du, Binyam Bezabeh, Ryan Fleming, Amanda Shannon, Shenlan Mao, Shannon Breen, Jing Zhang, Haihong Zhong, Jay Harper, Herren Wu, Philip W. Howard, Changshou Gao

**Affiliations:** 1 Antibody Discovery and Protein Engineering, MedImmune, Gaithersburg, MD 20878, USA; duq@medimmune.com (Q.D.); bezabehb@medimmune.com (B.B.); flemingr@medimmune.com (R.F.); amanda.shannon@marquette.edu (A.S.); wuh@medimmune.com (H.W.); 2 Spirogen Inc., London, E1 2AX, UK; tiberghiena@medimmune.com (A.C.T.); howardp@medimmune.com (P.W.H.); 3 Oncology Research, MedImmune, Gaithersburg, MD 20878, USA; maosh@medimmune.com (S.M.); breens@medimmune.com (S.B.); zhangj@medimmune.com (J.Z.); zhongh@medimmune.com (H.Z.); harperja@medimmune.com (J.H.)

**Keywords:** PBD dimer, thiosuccinimide hydrolysis, *N*-phenyl maleimide, retro-Michael reaction, serum stability, ADC, site-specific conjugation

## Abstract

Thiosuccinimide-linked antibody-drug conjugates (ADCs) are susceptible to drug loss over time due to a retro-Michael reaction, which can be prevented by selecting stable conjugation positions or hydrolysis of the thiosuccinimide. Here, we investigate pyrrolobenzodiazepine (PBD) ADC drug-linkers equipped with *N*-phenyl maleimide functionality for stable thiol conjugation via thiosuccinimide hydrolysis. Two PBD drug-linker formats (enzyme-cleavable and non-cleavable) were evaluated following site-specific conjugation to an engineered cysteine incorporated at position T289, which is known to be unstable for *N*-alkyl maleimide conjugates. *N*-phenyl maleimide PBDs conjugated to antibodies with similar efficiencies as *N*-alkyl maleimide PBDs and enhanced thiosuccinimide hydrolysis for *N*-phenyl maleimide PBDs was confirmed by mass spectrometry, capillary isoelectric focusing, and a SYPRO Orange dye binding assay. All of the PBD ADCs were highly potent in vitro regardless of maleimide- or linker-type, exhibiting low pM EC_50_ values. Thiol conjugation to *N*-phenyl maleimide PBD minimized the retro-Michael reaction in both rat and mouse serum. However, cleavage of the valine-alanine dipeptide in mouse serum for ADCs containing cleavable drug-linker led to drug loss regardless of maleimide type, which impacted ADC potency in tumor growth inhibition studies that were conducted in mouse models. Therapeutic improvement in mouse tumor models was realized for ADCs prepared with non-cleavable PBD drug-linkers that were conjugated through *N*-phenyl maleimide, where a stronger tumor growth inhibition (TGI) response was achieved when compared to the analogous *N*-alkyl maleimide drug-linker ADC. Altogether, our findings highlight the stability and efficacy benefits of *N*-phenyl maleimide functionality for ADCs that are produced with thiol-maleimide conjugation chemistry.

## 1. Introduction

Development of antibody-drug conjugates (ADCs) that stably link small molecule drugs to antibodies through efficient conjugation handles has resulted in much interest to expand conjugation technologies [1,2,3,4]. Thiol-maleimide coupling is often applied for production of ADCs because conjugates can easily be prepared through reaction with endogenous or engineered cysteine thiols [5,6]. Conjugation to endogenous thiols is achieved after the reduction of interchain disulfides of native antibodies, while site-specific conjugation is achieved by conjugation to engineered cysteines introduced into desired positions on antibody heavy or light chains.

Although thiol-maleimide coupling has the inherent advantages of fast reaction kinetics and excellent thiol-specificity, the resulting thiosuccinimide linkage is susceptible to the reverse reaction (retro-Michael addition), which results in drug loss from the antibody [7,8,9,10,11]. Drug loss impedes the ability of an ADC to deliver the maximum drug dose to the target site of activity and may also lead to undesired off-target effects, as released free drug will have different tissue distribution properties than the parent ADC. 

**Figure 1:**
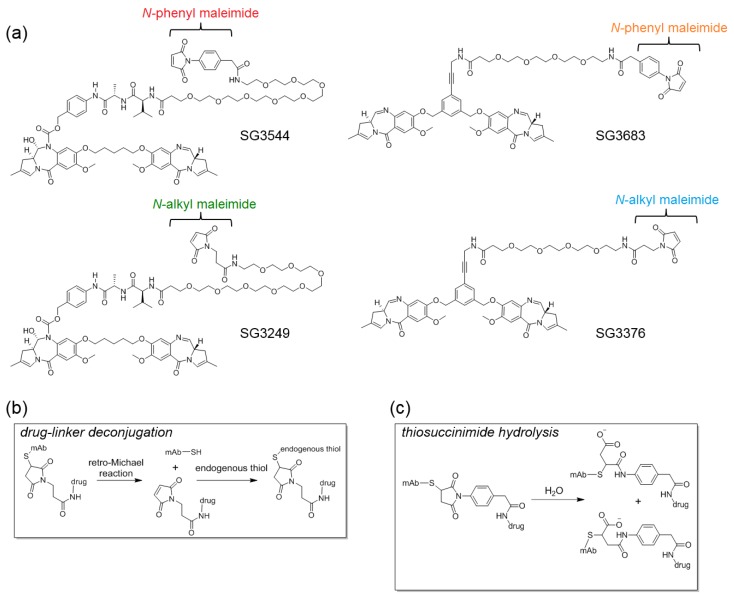
(**a**) Chemical structures of pyrrolobenzodiazepine (PBD) drug-linkers. Cleavable drug-linkers include SG3249 and SG3544 while non-cleavable drug-linkers include SG3376 and SG3683; (**b**) Overview of the retro-Michael deconjugation and thiol exchange process; and, (**c**) Ring-opening reaction of thiosuccinimide with water to generate stable thiol conjugates.

To circumvent the issue of thiol-maleimide conjugate instability, technologies have been developed that chemically prevent the retro-Michael reaction from occurring by hydrolyzing the thiosuccinimide that is formed after thiol-maleimide coupling (Figure 1). This can be achieved by designing maleimides with functional groups that facilitate spontaneous hydrolysis after conjugation, or, by subjecting an ADC to, conditions that accelerate the hydrolysis of thiosuccinimides formed with standard *N*-alkyl maleimides [9,12,13,14,15,16]. Functional groups that accelerate spontaneous thiosuccinimide hydrolysis and chemical stabilization of ADCs include; proximal amines, electron-withdrawing groups, and aromatic groups that are attached to the maleimide ring-head nitrogen [9,12,13,14,15,17]. Conditions that are employed to force the hydrolysis of *N*-alkyl thiosuccinimides that otherwise exhibit slow hydrolysis typically involve basic conditions, elevated temperature, or both [16]. Forced thiosuccinimide hydrolysis conditions must be carefully optimized to minimize the aggregation and degradation of the antibody and/or attached drug. Thus, maleimides that enable the spontaneous hydrolysis of thiosuccinimide conjugates offer a practical advantage since no additional treatment of ADCs is needed following the conjugation of drug-linker.

In addition to chemical methods, selecting appropriate cysteine conjugation positions on an antibody (introduced by protein engineering) can also significantly reduce drug-linker deconjugation. Such positions are typically buried within the monoclonal antibody (mAb) structure to prevent interactions with thiol-containing serum components, such as albumin, or are within a cationic microenvironment that accelerates thiosuccinimide hydrolysis [10,18]. Regardless of the approach used to improve conjugate stability of an ADC, stable constructs generally exhibit better tumor growth inhibition (TGI) in mouse tumor models [10,13]. The approach of chemical stabilization through maleimide design offers the benefit that conjugates will be stable regardless of the conjugation position on an antibody.

Here, we evaluate pyrrolobenzodiazepine (PBD) dimer ADC drug-linkers incorporating *N*-phenyl maleimide functionality that is aimed at improving the stability of the antibody-drug linkage. PBDs are a potent class of DNA active drugs that covalently crosslink DNA in a sequence-selective manner [19,20,21,22]. PBD dimers have evolved into potent ADC drug-linkers by incorporating conjugation handles for attachment to tumor-targeting antibodies and enzyme-cleavable linkers that enable drug release within target cells [23,24,25]. We previously evaluated the use of *N*-phenyl maleimide to stabilize thiol conjugates with the tubulin-active ADC drug-linker monomethyl auristatin E (MMAE) and reported second order conjugation rate constants, thiosuccinimide hydrolysis rate constants, retro-Michael deconjugation rate constants, and serum stability [12]. However, there is currently no DNA-active ADC drug-linkers that are equipped with stable thiol conjugation functionality. Thus, we installed *N*-phenyl maleimide onto PBD drug-linkers and studied the physicochemical properties and biological activity of stable ADCs targeting a different mechanism of action than previously reported tubulin-active ADCs.

*N*-phenyl maleimide PBD drug-linker analogues of SG3249 (with an enzyme cleavable dipeptide linker) and SG3376 (with a non-cleavable linker) were synthesized for this work and named SG3544 and SG3683, respectively [25]. All of the PBD drug-linkers produced high quality ADCs (high conjugation efficiency and low aggregation) when conjugated to a solvent exposed engineered cysteine introduced by mutation of position T289 of anti-5T4 antibody (termed A07-108-T289C). Position T289C is known to produce unstable thiol-*N*-alkyl maleimide conjugates, and thus serves as a model to assess the ability of *N*-phenyl maleimide to prevent deconjugation. Enhanced thiosuccinimide hydrolysis for *N*-phenyl maleimide conjugates was determined by mass spectrometry, capillary isoelectric focusing (CIEF), and SYPRO Orange dye binding assays. Conjugate stability in mouse and rat sera indicated that *N*-phenyl maleimide drug-linkers contained a more stable thiol linkage, but enzymatic cleavage of the valine-alanine (val-ala) dipeptide spacer in SG3249 and SG3544 drug-linkers in mouse serum at the highly exposed T289C position led to significant drug loss. Non-cleavable PBD drug-linkers SG3376 and SG3683 did not exhibit linker cleavage in mouse serum and an increased drug loss for SG3376 following serum incubation was directly attributed to the retro-Michael reaction. The benefit of stable thiol conjugation was demonstrated by stronger TGI activity in murine xenograft tumor model efficacy studies for ADC that was prepared with the non-cleavable PBD drug-linker SG3683, confirming that thiol conjugation of PBD drug-linker via *N*-phenyl maleimide can lead to improved performance in vivo.

## 2. Materials and Methods

### 2.1. General

All of the PBD drug-linkers were provided by Spirogen, Inc., London, UK, a member of the AstraZeneca group. SG3544 was prepared from a common intermediate described in our previous synthesis of SG3249 [25]. SG3376 and SG3683 were prepared using methods described in our prior patent application and recently manuscripts [26,27,28]. Synthesis details for installing *N*-phenyl maleimide functionality onto PBD drug-linkers is provided in the Supporting Information section. Prior to conjugation experiments, 10 mM drug-linker stock solutions were prepared in dimethyl sulfoxide (DMSO) for SG3249 and SG3376 or dimethyl acetamide (DMAc) for SG3544 and SG3683. Antibodies were prepared using standard molecular biology methods.

### 2.2. Preparation of ADCs

ADCs were prepared by site specific conjugation of PBD drug-linkers to cysteine-engineered antibodies comprising a T289C mutation. A representative protocol for site-specific conjugation is as follows: First, antibody solution (80 mL of 3.13 mg/mL solution in phosphate buffered saline (PBS), 1.7 µmol, 1 eq.) was combined with TCEP (1.33 mL of a 50 mM solution in water, 67 µmol, 40 eq.), followed by gentle mixing at 37 °C for 1 h. Reduced antibody was transferred to a slide-a-lyzer dialysis cassette (10 K MWCO) and dialyzed against PBS, 1 mM EDTA, pH 7.4, 4 °C for 24 h with several buffer changes. Reduced antibody was oxidized to reform internal disulfides by the addition of dehydroascorbic acid (667 µL of 50 mM stock in DMSO, 33 µmol, 20 eq.), followed by incubation for 4 h at room temperature. Oxidized antibody solution (70.4 mL of 3.41 mg/mL, 1.6 µmol, 1 eq.) was combined with 14 mL DMSO (final concentration 20% *v*/*v*), followed by the addition of PBD drug-linker (800 µL of a 10 mM solution, 8 µmol, 5 eq.). The reaction proceeded at room temperature with mixing for 1 h followed by addition of *N*-acetyl cysteine (640 µL of 100 mM stock in water, 64 µmol, 40 eq.) to quench unreacted maleimide. The reaction mixture was then diluted 3-fold with distilled water and subjected to CHT chromatography to remove unconjugated PBD (CHT Type II 40 µm media). ADC was eluted with a gradient from buffer A (10 mM phosphate, pH 7.0) to buffer B (10 mM phosphate pH 7.0 containing 2 M NaCl) over 25 min at a flow rate of 5 mL/min. After CHT chromatography, ADC samples were buffer exchanged to PBS, pH 7.4 by dialysis in a slide-a-lyzer cassette (20 K MWCO) at 4 °C for 24 h with four buffer changes. Typical yield: 180–200 mg, 75–83%. This general procedure was used for site-specific conjugation of all PBD drug-linkers, with only the amount of starting antibody being varied, all of the other ratios and equivalents remained the same. Two different human IgG1 antibodies were used to generate ADCs: A07-108 (anti-5T4) and a non-binding isotype control (termed IgG).

### 2.3. Mass Spectrometry

Antibody conjugates were deglycosylated and reduced prior to analysis. ADC samples were diluted to 0.2 mg/mL with PBS pH 7.4 and then 50 µL of antibody solution was combined with 1.5 μL of Remove-iT^®^ EndoS solution (200 K units/mL, New England Biolabs, Ipswich, MA, USA) and 5 µL 10× Glyco Buffer 1 (New England Biolabs). Deglycosylation proceeded at 37 °C for 1 h followed by addition of 5 µL TCEP (0.5 M in water) and further incubation for 5 min at 37 °C to reduce antibody disulfides.

Mass spectrometry (LC/MS) analysis was performed using an Agilent 6520B Q-TOF mass spectrometer equipped with a reverse phase high performance liquid chromatography (RP-HPLC) column (Agilent Poroshell 300SB-C3; 5 µm, 2.1 mm × 75 mm, Santa Clara, CA, USA). RP-HPLC parameters were as follows: injection volume: 15 μL, flow rate, 0.4 mL/min; mobile phase A was 0.1% (*v*/*v*) formic acid in HPLC-grade H_2_O; and, mobile phase B was 0.1% (*v*/*v*) formic acid in acetonitrile. The column was equilibrated in 90% A/10% B, which was also used to desalt the ADC samples, followed by elution in 40% A/60% B. Mass data were collected for 100–3000 *m*/*z*, positive polarity, a gas temperature of 350 °C, a nebulizer pressure of 48 lb/in^2^, and a capillary voltage of 5000 V. Data were analyzed using vendor-supplied (Agilent v.B.04.00, Santa Clara, CA, USA) MassHunter Qualitative Analysis software and peak intensities from deconvoluted spectra were used to derive the relative proportion of species (conjugated mAb, free mAb and hydrolyzed thiosuccinimide) in each sample, as previously described [12,13,16]. For ADC samples with low thiosuccinimide hydrolysis (i.e., comprising *N*-alkyl maleimide drug-linkers), resolution of the sodium adducts (+22 amu) from hydrolyzed species (+18 amu) was difficult. In this case, thiosuccinimide hydrolysis in ADC samples was estimated by subtracting out the relative sodium adduct abundance, as observed for the parent A07-108 antibody. 

### 2.4. rRP-HPLC

ADCs were reduced at 37 °C for 20 min in the presence of 42 mM dithiothreitol (DTT) in PBS pH 7.2. Reduced sample (10 µg) was loaded onto a PLRP-S, 1000 Å column (2.1 × 50 mm, Agilent, Santa Clara, CA, USA) and eluted at 80 °C at a flow rate of l mL/min with a gradient of 5% B to 100% B over 25 min (mobile phase A: 0.1% trifluoroacetic acid in water, and mobile phase B: 0.1% trifluoroacetic acid in acetonitrile). Percent conjugation was determined using integrated peak areas from the chromatogram.

### 2.5. Capillary Isoelectric Focusing Immunoassay (cIEF)

Antibody and ADC samples were analyzed on a Peggy Sue instrument (NanoPro, San Jose, CA, USA), according to manufacturer’s instructions from ProteinSimple. Briefly, mAb and ADCs were diluted to 4 µg/mL in Simple Dilute (ProteinSimple, San Jose, CA, USA). Samples were combined with Premix G2 pH separation gradient containing fluorescence labeled pI standards (pI 4, 5.5, 7.3, 8.4, and 9.7e), 0.25% TEMED (Amresco, Solon, OH, USA), 50% Premix G2 pH 4–10 (ProteinSimple) and 4% of pH 8–10.5 Pharmalytes (GE Health Sciences, Pittsburgh, PA, USA). The detection anti-human IgG Fc-HRP conjugated mouse antibody (Jackson ImmunoResearch, West Grove, PA, USA) was diluted 1:200 into Antibody Diluent (ProteinSimple). Luminol and Peroxide-XDR (ProteinSimple) was mixed at 1:1 ratio and were used as a chemiluminescent substrate. The samples (12 µL/well) and detection reagents (20 µL/well) were loaded into a 384-well assay plate (ProteinSimple), followed by centrifugation at 3000× *g* for 5 min at 4 °C to remove air bubbles in the plate. The 384-well simple plate, charge separation capillary box, and buffers were loaded into a Peggy Sue instrument, according to the user’s manual. Automated separation and detection were performed using the default charge separation settings. Capillaries were imaged and analyzed with Compass software (ProteinSimple) and pI values were determined by distance traveled in the capillary relative to controls.

### 2.6. SYPRO Orange Binding Assay

Antibody or ADC solutions (25 µL, 1 mg/mL in PBS) were combined with 50X SYPRO Orange solution (2.5 µL, diluted from 5000X stock with water, Invitrogen, Carlsbad, CA, USA) in a 96-well RT-PCR plate. Samples were then loaded into a CFX96 Real-Time System equipped with a C1000 Thermal Cycler instrument (BioRad, Hercules, CA, USA) and the temperature was equilibrated to 20 °C for 10 min. Fluorescence intensity was measured at 20 °C and the fluorescence intensity is reported as the average measurement value of five samples ± standard deviation.

### 2.7. ADC Serum Stability

ADCs were incubated in mouse and rat sera to challenge the stability of the thiosuccinimide linkage. ADC samples were added to normal mouse or rat serum (Jackson Immunoresearch, West Grove, PA, USA) to achieve a final concentration of 0.2 mg/mL (1.33 µM antibody), with the total volume of ADC solution added to serum being less than 10%. The ADC-serum mixture was sterile filtered and incubated at 37 °C for seven days in a sealed container without stirring. Total human antibody (PBD-conjugated and unconjugated) was recovered from serum by immunoprecipitation using Fc-specific anti-human IgG-agarose resin (Sigma-Aldrich, St. Louis, MO, USA). Resin was rinsed twice with PBS, once with IgG elution buffer, and then twice more with PBS. ADC-containing serum samples were then combined with anti-human IgG resin (100 µL of ADC-serum mixture, 50 µL resin slurry) and gently mixed for 15 min at room temperature. Resin was recovered by centrifugation and then washed twice with PBS. The resin pellet was resuspended in 100 µL IgG elution buffer (Thermo Scientific, Waltham, MA, USA) and further incubated for 5 min at room temperature. Resin was removed by centrifugation and then 20 µL of 10X glycol buffer 1 (New England Biolabs, Ipswich, MA, USA) was added to the supernatant. Recovered human antibody solution was sterile filtered and deglycosylated by adding 1.5 uL Remove-iT^®^ EndoS (200K units/mL, New England Biolabs, Ipswich, MA, USA), and followed by further incubation at 37 °C for 1 h. Deglycosylated ADCs were reduced with TCEP and analyzed by LC/MS. Percent conjugated antibody was determined from peak heights of mass spectra similar to a previously described method [29].

### 2.8. Cytotoxicity Analysis

Site-specific A07-108 T289C ADCs were evaluated for in vitro potency against receptor positive MDA-MB-361 breast cancer cells. Cells were plated in 80 µL of Leibovitz’s L-15 culture medium containing 20% FBS into 96-well flat-bottomed plates at 5000 cells/well. Cells were allowed to adhere overnight. A 5X concentration stock solution of each ADC was prepared by diluting the test articles in culture medium. Twenty microliters of each test article were added to cells in duplicate in a stepwise 1:4 serial dilution series. Treated cells were cultured at 37 °C/0% CO_2_ for six days, and cell viability was assessed with the Cell Titer-Glo (CTG) Luminescent Viability Assay from Promega. 100 µL of reconstituted CTG reagent was added to each well and the plate was mildly shaken for 10 min at room temperature. The luminescence of each sample at 560 nm was read using a Perkin Elmer EnVision luminometer (Waltham, MA, USA). Percent cell viability was calculated by the following formula: (average luminescence of treated samples/average luminescence of untreated control samples) × 100. EC_50_ values were determined using logistic non-linear regression analysis with GraphPad Prism v7.02 software (La Jolla, CA, USA).

### 2.9. Tumor Growth Inhibition

All of the animal procedures were performed in accordance with appropriate regulatory standards under protocols approved by the MedImmune Institutional Animal Care and Use Committee. In vivo efficacy studies were performed using five- to six-week-old female athymic nude mice (Harlan Sprague Dawley Inc., Indianapolis, IN, USA). Sixty day 0.36 mg slow release estradiol pellets were implanted subcutaneously into the dorsal flank of mice the day before tumor cell inoculation. Ten million MDA-MB-361 cells in 50% Matrigel were injected subcutaneously into the 2nd mammary fat pad of mice to generate tumors. When tumors reached approximately 200 mm^3^ mice were randomized based upon tumor volume and assigned into groups (*n* = 10, each group). IgG control- or A07-108-PBD ADCs were administered at 0.3 or 1 mg/kg intravenously. Tumors were measured twice weekly with calipers and tumor volumes were calculated using the formula:

V = ½ × L × W^2^ (1)

where L = length; W = width. Tumor growth graphs were plotted using GraphPad Prism v7.02 software (La Jolla, CA, USA). Tumor volumes are expressed as mean ± standard deviation. Indicated *p* values were determined by the Student’s *t* test using a two-tailed distribution and two-sample unequal variance with the *t*-test function of Microsoft Excel. The *p* values of less than 0.05 were considered as statistically significant.

## 3. Results

### 3.1. ADC Preparation and Characterization

**Figure 2:**
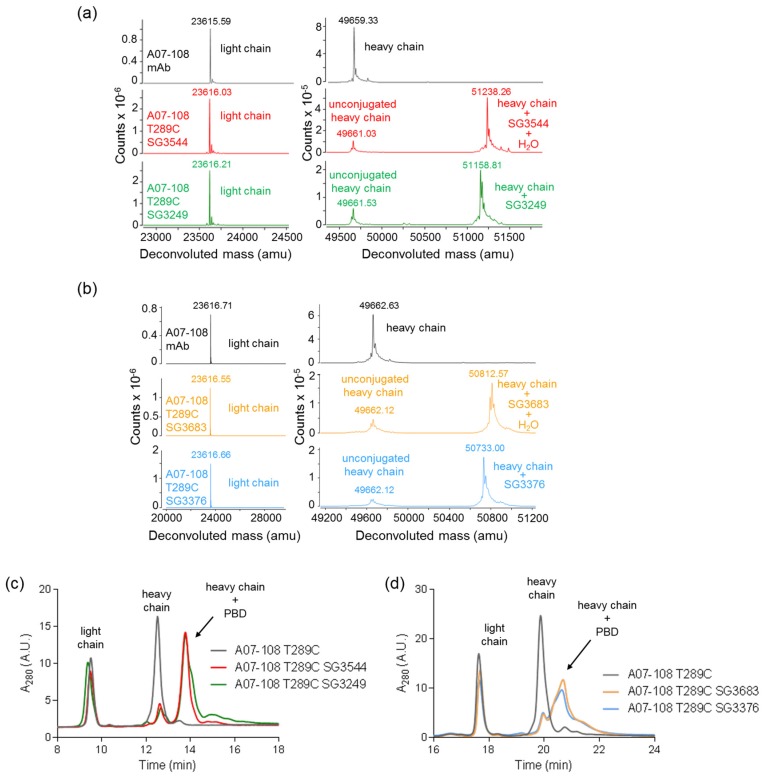
Characterization of A07-108 T289C antibody-drug conjugates (ADCs). (**a**) Reduced deglycosylated mass spectrometry analysis of antibody before and after conjugation of cleavable pyrrolobenzodiazepine (PBD) drug-linkers SG3249 and SG3544; (**b**) Reduced deglycosylated mass spectrometry analysis of antibody before and after conjugation of non-cleavable PBD drug-linkers SG3376 and SG3683; (**c**) rRP-HPLC analysis of antibody before and after conjugation to cleavable PBD drug linkers SG3249 and SG3544; and, (**d**) rRP-HPLC analysis of antibody before and after conjugation to non-cleavable drug-linkers SG3376 and SG3683. Antibody light chain, unconjugated heavy chain and conjugated heavy chain are indicated. Spectra are zoomed in to show light chain and heavy chain regions in both (**a**,**b**).

Conjugation efficiencies to mAb position T289C were similar for all four PBD drug-linkers, with 84–88% conjugated antibody observed and the expected mass of each drug-linker was confirmed by mass spectrometry (Figure 2 and Table 1). Both mass spectrometry and rRP-HPLC demonstrated that conjugation was specific to the antibody heavy chain, as expected since the T289C mutation is contained on the antibody heavy chain (Figure 2 and Figure S1). Mass spectrometry analysis also indicated that the majority (75–80%) of thiosuccinimides hydrolysized during the conjugation and purification process (CHT chromatography and dialysis) in the case of ADCs conjugated with either SG3544 or SG3683, whereas less hydrolysis occurred for ADCs that were conjugated with SG3249 or SG3376 (30–36%) (Figure 2a,b). Finally, size exclusion chromatography analysis showed that all ADC preparations contained greater than 90% monomer (Table 1, Figure S2). Thus, *N*-phenyl maleimide drug-linkers did not affect the conjugation efficiency or the propensity of ADCs to aggregate compared to *N*-alkyl maleimide drug-linkers.

### 3.2. Evaluation of Thiosuccinimide Hydrolysis by CIEF and SYPRO Orange Binding

**Figure 3:**
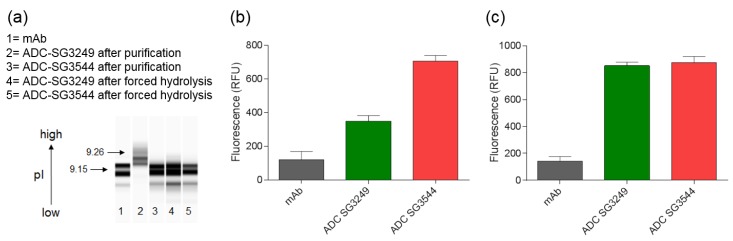
Evaluation of thiosuccinimide hydrolysis by capillary isoelectric focusing (CIEF) measurement and SYPRO Orange fluorescence. (**a**) CIEF analysis of monoclonal antibody (mAb) and antibody-drug conjugates (ADCs) before and after thiosuccinimide hydrolysis; (**b**) Fluorescence intensity of mAb and ADCs upon incubation with SYPRO Orange dye; and, (**c**) Fluorescence intensity of mAb and ADCs upon incubation with SYPRO Orange dye after thiosuccinimide hydrolysis. Thiosuccinimide hydrolysis was achieved by incubation at 37 °C, pH 7.8, three days for samples in (**a**,**c**).

Thiosuccinimide hydrolysis was investigated via capillary isoelectric focusing (CIEF), which revealed a shift in the isoelectric point (pI). Thiosuccinimide hydrolysis lowered the pI of ADCs, as evidenced by the difference between A07-108 T289C SG3544 (mostly hydrolyzed) and A07-108-T289C SG3249 (mostly unhydrolyzed) ADCs (Figure 3). Conjugation of PBD drug-linker resulted in an increase (~0.11 units) in pI for A07-108-SG3249 when compared to unconjugated mAb, probably due to protonatable nitrogens in the PBD drug-linker molecule. However, this increase in pI was not observed for the A07-108-SG3544 ADC, which is isolated primarily as the hydrolyzed product after conjugation and purification. Both of the ADCs were subjected to a “forced hydrolysis” procedure (incubation at pH 7.8, 37 °C for three days, complete hydrolysis confirmed by mass spectrometry), which lowered the pI of the A07-108-T289C-SG3249 sample to the same value of A07-108-T289C-SG3544, which itself remained unchanged.

Thiosuccinimide hydrolysis was also evaluated using a SYPRO Orange dye binding assay. SYPRO Orange fluoresces upon binding to hydrophobic patches on proteins and is used to determine melting temperatures as they denature and bind dye [30,31,32,33]. As shown in Figure 3b, the A07-108-T289C SG3544 ADC exhibited higher SYPRO Orange dye binding immediately after purification when compared to the analogous SG3249 ADC. After subjecting both ADCs to thiosuccinimide hydrolysis conditions (incubation at pH 7.8, 37 °C for three days), they both showed the same fluorescence intensity, indicating that thiosuccinimide hydrolysis was complete (Figure 3c). Comparison of SYPRO fluorescence for the SG3544 construct before and after the forced hydrolysis procedure demonstrates that ~80% of thiosuccinimide groups were hydrolyzed in the ADC product that was obtained after purification, which is consistent with mass spectrometry results.

### 3.3. Serum Stability of ADCs

Thiosuccinimide linkage stability was evaluated by subjecting ADCs to incubation in reconstituted mouse and rat serum (37 °C, 7 days). PBD loss was determined following immunocapture of human mAbs/ADCs from serum and mass spectrometry analysis, similar to previously described methods [29].

**Figure 4:**
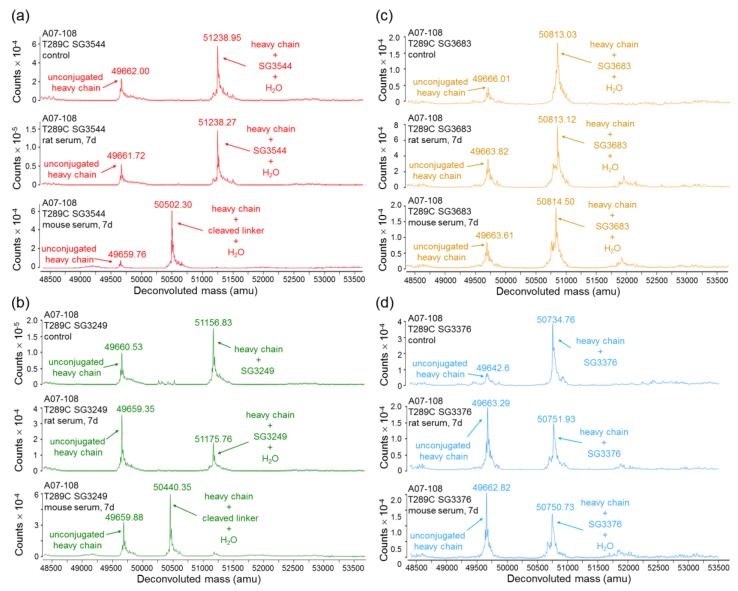
Mass spectrometry analysis of serum-incubated ADCs. (**a**) A07-108-T289C SG3544; (**b**) A07-108 T289C SG3249; (**c**) A07-108-T289C SG3683; and, (**d**) A07-108-T289C SG3376. All of the samples were analyzed immediately after addition to serum (control), after incubation in rat serum for seven days at 37 °C, and after incubation in mouse serum for seven days at 37 °C. All of the spectra are zoomed in to show the heavy-chain only.

**Figure 5:**
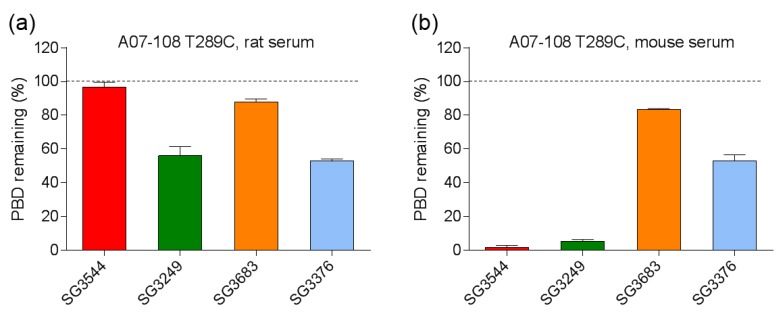
Quantification of pyrrolobenzodiazepine (PBD) loss from antibody-drug conjugates (ADCs) following incubation in (**a**) rat and (**b**) mouse serum at 37 °C for seven days. Remaining PBD was determined by mass spectrometry. Only intact PBD drug-linker was analyzed, linker-cleaved species were not included in the analysis.

Incubation of ADCs in rat serum confirmed improved thiosuccinimide stability for *N*-phenyl maleimide PBD drug-linkers as compared to analogous *N*-alkyl maleimide PBD drug-linkers (Figure 4 and Figure 5). Additionally, no val-ala linker cleavage was observed following incubation in rat serum, which resulted in >80% retention of intact drug-linker for SG3544 and SG3683 ADCs. ADCs prepared with the *N*-alkyl maleimide PBD drug-linkers SG3249 and SG3376 more (~45%) drug loss. Further examination of mass spectrometry results for *N*-alkyl maleimide conjugates SG3249 and SG3376 (Figure 4b,d) confirmed that the drug-linker species remaining after seven days incubation in rat serum was the thiosuccinimide-hydrolyzed species as expected (evidenced by +18 amu relative to control). We previously determined retro-Michael deconjugation as a function of time in mouse serum, and showed that thiosuccinimide hydrolysis limits drug-linker deconjugation [12]. Thus, the seven-day experiment in the current study represents an endpoint measurement as no further deconjugation by the retro-Michael mechanism is expected since thiosuccinimides were completely hydrolyzed.

Mouse serum stability results indicated the nearly complete loss of PBD drug from ADCs that were prepared with the cleavable drug-linkers SG3249 and SG3544, regardless of maleimide type (Figure 4 and Figure 5). This outcome was affected primarily by cleavage of the val-ala dipeptide spacer in the drug-linker, which left only the linker attached to the mAb (Figure 4a,b and Figure S3). When the degraded linker is considered as a conjugated species, >90% conjugation (i.e., intact thiol linkage) was retained for the *N*-phenyl maleimide SG3544 ADC, whereas the *N*-alkyl maleimide SG3249 ADC showed ~35% loss of conjugation. Non-cleavable PBD drug-linkers (SG3376 and SG3683) were not impacted by dipeptide cleavage in mouse serum and drug loss could be solely attributed to the retro-Michael reaction deconjugation mechanism. A07-108-T289C SG3376 ADC lost ~45% PBD and A07-108-T289C SG3683 lost ~15% PBD over the seven days incubation period in mouse serum (Figure 5).

### 3.4. In Vitro Activity of ADCs

**Figure 6:**
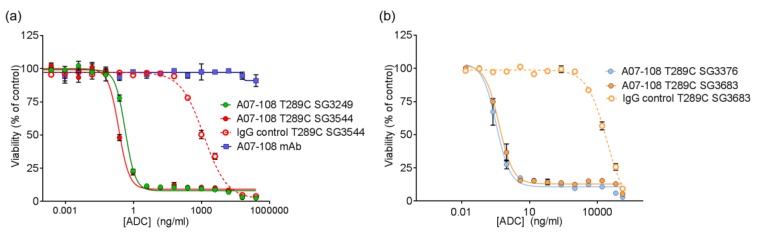
In vitro cytotoxicity of A07-108 antibody-drug conjugates (ADCs) towards cultured MDA-MB-361 cells. (**a**) Activity of ADCs prepared with cleavable pyrrolobenzodiazepine (PBD) drug-linkers; and (**b**) activity of ADCs prepared with non-cleavable PBD drug-linkers.

The cytotoxicity of ADCs conjugated with either *N*-alkyl maleimide or *N*-phenyl maleimide PBD drug-linkers towards target-positive cultured cancer cell lines was evaluated. Non-targeting ADCs were also included as isotype controls. A07-108-T289 ADCs demonstrated potent cytotoxicity of their respective target-positive cells, with single digit pM EC_50_ values observed for all of the PBD drug-linkers (Figure 6, Table 2). These observed potencies are similar to the value previously reported for an ADC prepared with SG3249, which also exhibited single digit pM EC_50_ values in cytotoxicity assays [25]. There was no difference in activity between ADCs that were prepared with cleavable (SG3249 and SG3544) and non-cleavable (SG3376 and SG3683) drug-linkers, indicating that non-specific degradation of the antibody following internalization of the ADC was sufficient to release active drug. Further studies are necessary to elucidate the mechanism of PBD release and subcellular trafficking in the case of non-cleavable drug-linkers described here [34].

### 3.5. In Vivo Activity of ADCs

**Figure 7:**
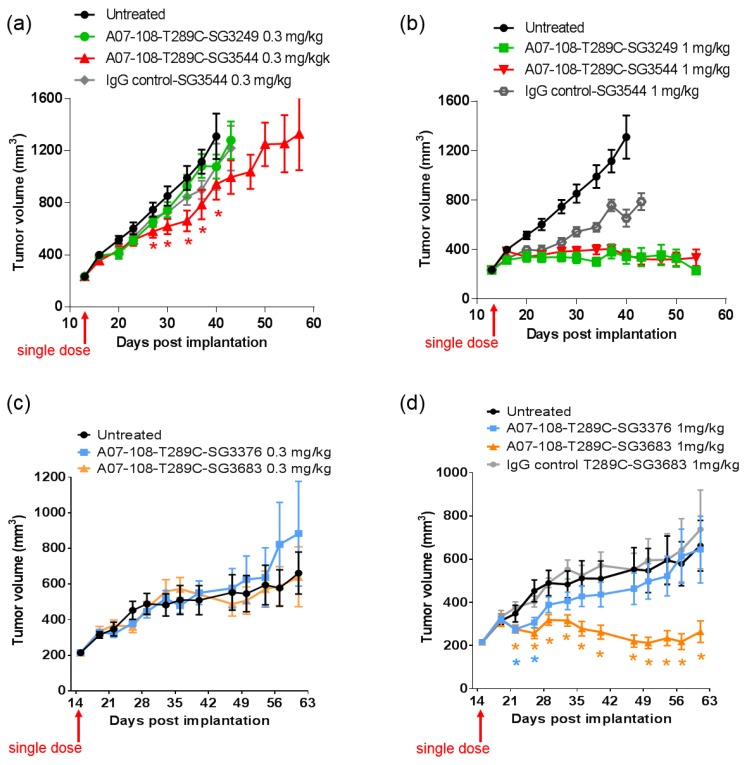
Tumor growth inhibition of MDA-MB-361 tumor models in mice following treatment with A07-108 antibody-drug conjugates (ADCs). ADCs were administered by a single dose I.V. as indicated. (**a**) Cleavable ADCs dosed at 0.3 mg/kg; (**b**) cleavable ADCs dosed at 1 mg/kg; (**c**) Non-cleavable ADCs dosed at 0.3 mg/kg; (**d**) Non-cleavable ADCs dosed at 1 mg/kg. Data represents the average tumor volume ± standard deviation, *n* = 10. Statistical difference (*p* < 0.05) between ADC-treated groups and non-treated controls are indicated with * symbols in (**a**,**d**) where * symbols are colored to match the corresponding curve color.

ADCs prepared with the A07-108-T289C mAb conjugated with the PBD drug-linkers were evaluated in mouse xenograft MDA-MB-361 tumor models that express high levels of 5T4 [35]. Mice were administered a single dose of ADC by tail-vein injection and tumor growth inhibition was monitored over time. ADCs bearing the cleavable PBD drug-linkers SG3249 or SG3544 both achieved tumor stasis following a single I.V. administration at 1 mg/kg, however activity was much reduced when dosed at 0.3 mg/kg. At the 0.3 mg/kg dose, ADC conjugated with SG3249 was ineffective against the MDA-MB-361 tumors, whereas modest tumor growth inhibition (TGI) was observed for SG3544 ADC. For the SG3544 group, tumors were statistically smaller (*p* < 0.05) than tumors in the non-treated group from days 27–40 (Figure 7a). A statistically significant decrease in average tumor volume was not achieved for the SG3249 group relative to non-treated controls at any point in the study period. The small difference in TGI observed at 0.3 mg/kg for the SG3544 group suggests at least a minor improvement in ADC performance due to stable thiol conjugation of drug-linker via *N*-phenyl maleimide, however, neither tumor stasis nor regression was achieved and the improvement was not as clear as desired. At the 1 mg/kg dose, there was no difference in TGI of ADCs conjugated with cleavable PBD drug-linkers SG3249 or SG3544. Significant tumor stasis was at the 1 mg/kg dose made it difficult to determine the differences in activity (Figure 7b).

ADCs prepared with non-cleavable PBD drug-linkers showed no antitumor activity when dosed at 0.3 mg/kg (Figure 7c). At 1 mg/kg, tumor stasis and clear differentiation relative to non-treated controls (*p* < 0.05) was achieved with A07-108-T289C SG3683 ADC and no TGI was observed for A07-108-T289C SG3376 ADC, demonstrating the therapeutic benefit for stable thiol conjugation through *N*-phenyl maleimide (Figure 7d). Statistical difference (*p* < 0.05) between ADC-treated and non-treated control groups for SG3683 was observed at 22 days, and was maintained for the duration of the study. The SG3376 group showed statistical difference from the non-treated control group only at days 22 and 26, after which the average tumor size was not significantly different than non-treated controls.

## 4. Discussion

The reactive group utilized for conjugating drug-linkers to antibody cysteines is typically an *N*-alkyl maleimide, which couples to thiols via a Michael-addition reaction [23,24,25]. The reversible nature of the Michael reaction can lead to deconjugation and the loss of drug from the antibody over time (depending on the conjugation position), which can be prevented by post-conjugation thiosuccinimide hydrolysis [9,12,13,14,16]. To date, maleimides that are engineered to facilitate post-conjugation thiosuccinimide hydrolysis have been installed onto the tubulin-inhibiting drug monomethyl auristatin E (MMAE) [13]. This toxin is fairly accommodating to different functional groups that aid in thiosuccinimide hydrolysis, as there are no reactive or chemically sensitive groups present. PBDs, however, contain an imine functional group that is not compatible with nucleophilic functional groups (such as primary amines) used to assist thiosuccinimide hydrolysis. Thus, we chose to incorporate the *N*-phenyl maleimide functional group into PBD ADC drug-linkers to generate a new class of stably conjugated DNA-active ADCs. *N*-phenyl maleimides produce thiosuccinimide conjugates that spontaneously hydrolyze under ambient conditions without any special treatment [12]. Cleavable and non-cleavable PBD drug-linkers were equipped with *N*-phenyl maleimides and compared to analogous *N*-alkyl maleimide PBD drug-linkers.

ADCs were synthesized by site-specific thiol conjugation of PBD drug-linkers to position 289 where a cysteine had been engineered to replace the native threonine (T289C). Position T289C was selected as a model conjugation site due to the known instability of *N*-alkyl maleimide cysteine conjugates, thus allowing for the stabilization of conjugates by *N*-phenyl maleimide to be assessed by direct comparison with analogous *N*-alkyl maleimide conjugates [12]. Conjugation to a highly solvent-exposed position allows for clear differences in stability to be observed, however, additional drug loss due to species-specific enzymatic activity (i.e., mouse) must also be considered. Enzymatic cleavage of common dipeptide linkers in mouse serum is a known property of ADCs that are prepared with Cathepsin B substrates (as was observed here). However, intact thiol linkages (hydrolyzed or non-hydrolyzed thiosuccinimides) can be tracked by mass spectrometry, and thus confirm stable thiol conjugation despite degradation of drug-linker at other sites. Altogether, stable thiol-maleimide conjugation at position T289C with *N*-phenyl maleimide PBD demonstrates the potential to chemically prevent drug-linker deconjugation at a non-ideal position for *N*-alkyl maleimides.

Site-specific conjugation to T289C was achieved by first reducing the antibody to generate free sulfhydryls, followed by dialysis to remove reducing agent, then mild oxidation to reform hinge disulfides, and finally the addition of drug-linker. Mild reduction is necessary for site-specific conjugation because engineered cysteines are often capped with a cysteine amino acid (as a disulfide) in the isolated antibody product. Free sulfhydryl was generated by disulfide reduction, and liberated cysteine was removed by dialysis since it will also react with maleimide and decrease conjugation efficiency. Finally, native disulfides must be reformed in the antibody by the oxidation of sulfhydryls with dehydroascorbic acid to prevent the conjugation to off-target cysteines, resulting in an inhomogeneous product.

One concern regarding hydrolysable maleimides is that they could hydrolyze and become inactivated prior to reaction with cysteine thiols, thus reducing conjugation efficiency. However, this was not the case as *N*-phenyl maleimide PBD drug-linkers conjugated with similar efficiency as *N*-alkyl maleimide PBD drug-linkers. This result is consistent with our previous work with demonstrating efficient conjugation of a *N*-phenyl maleimide MMAE to antibodies [12]. High conjugation efficiency is maintained since cysteine-maleimide coupling occurs much faster than *N*-phenyl maleimide hydrolysis, thus minimizing the impact on conjugation.

Post-conjugation thiosuccinimide hydrolysis is necessary for chemical stabilization of the thiol-drug linkage in ADCs that are prepared with maleimide drug-linkers [12,13,14,16]. Mass spectrometry analysis of ADCs following CHT purification revealed that the majority (80–86%) of *N*-phenyl thiosuccinimides were hydrolyzed for SG3544 and SG3683 drug-linker ADCs, as indicated by +18 amu in addition to the drug-linker mass. *N*-phenyl thiosuccinimide hydrolysis occurred spontaneously during the conjugation and purification process (CHT chromatography and 24 h dialysis) without any special treatment. *N*-alkyl thiosuccinimide hydrolysis of SG3249 and SG3376 was also observed (30–36%), although at a much lower prevalence than *N*-phenyl thiosuccinimide hydrolysis in SG3544 and SG3683. The precise determination of low degrees of thiosuccinimide hydrolysis is difficult due to interference of the sodium adduct species (+22 amu) present in all of the antibody species that were analyzed by mass spectrometry, thus the degree of thiosuccinimide hydrolysis for SG3249 and SG3376 should be considered as an estimate. Ideally, samples prepared with 100% (*N*-phenyl) and 0% (*N*-alkyl) hydrolyzed thiosuccinimide would allow for the maximum effect of thiosuccinimide hydrolysis to be observed. However, thiosuccinimide hydrolysis occurs spontaneously during purification and cannot be eliminated completely in the case of *N*-alkyl maleimide conjugates. *N*-phenyl thiosuccinimides in SG3376 and SG3683 ADCs were not intentionally hydrolyzed further, as one of the benefits of *N*-phenyl maleimides is their ability to hydrolyze without any special treatment. Thus, we chose to proceed with the ADCs that were obtained under standard conjugation and purification procedures.

To date, mass spectrometry has been the most utilized analytical method to determine thiosuccinimide hydrolysis, since the addition of 18 amu can be directly observed. Detection of thiosuccinimide hydrolysis by other methods should also be possible, since an anionic carboxyl group is formed. Indeed, CIEF analysis confirmed the introduction of carboxyl groups, as evidenced by reduction of pI. For the A07-108-T289C SG3544 ADC evaluated here, the reduction in pI was modest when compared to the overall pI of the ADC. However, the impact of thiosuccinimide hydrolysis could be antibody dependent, as introduction of anionic groups into antibodies with lower initial pI values (i.e., near 7) might result in a greater pI drop than observed for the A07-108-T289C mAb evaluated here (initial pI = 9.15). The biological effect of this decrease in ADC pI is unknown at present, however, lower pI could affect interactions of ADCs with charged components in serum or tissues following administration and impact properties, such as pharmacokinetics and biodistribution [36,37].

SYPRO Orange binding also demonstrated a difference in ADCs containing mostly hydrolyzed or mostly unhydrolyzed thiosuccinimides. The use of SYPRO Orange to detect the generation of carboxylates in hydrolyzed ADC drug-linker has not been reported previously, as the standard use of this dye is to determine protein melting temperatures. SYPRO Orange is a useful bioanalytical tool because it binds to hydrophobic patches and fluoresces as a protein melts, thus fluorescence as a function of temperature can be measured. Analysis of SYPRO Orange binding to ADCs in the current work demonstrated that drug-linkers generate an artificial hydrophobic patch on antibodies, as was reflected in the 2–4-fold increase in fluorescence for ADCs when compared to unconjugated mAb. When considering that SYPRO Orange is reported to have two amines and one sulfate group in its structure, which would result in a net cationic molecule, we hypothesized that thiosuccinimide hydrolysis would increase dye binding to an ADC due to the generation of anionic carboxylates in close proximity to a hydrophobic drug-linker [38]. Indeed, increased SYPRO Orange dye binding to ADCs with hydrolyzed thiosuccinimides was observed, demonstrating that SYPRO Orange can be used to detect the introduction of anionic carboxylate groups in ADC drug-linkers. Altogether, SYPRO Orange binding results corroborated well with CIEF measurements, confirming that ADCs comprising *N*-phenyl thiosuccinimides are isolated primarily as the hydrolyzed product and both *N*-alkyl and *N*-phenyl thiosuccinimide ADCs behave similarly after the forced hydrolysis procedure. Altogether, CIEF and SYPRO Orange data confirm that thiosuccinimide hydrolysis results in a detectable change in the physical properties of an ADC, and that thiosuccinimides formed with *N*-phenyl maleimide SG3544 drug-linker undergo hydrolysis more efficiently than those that are formed with *N*-alkyl maleimide SG3249 drug-linker.

Serum stability evaluation of ADCs confirmed that *N*-phenyl maleimide drug-linkers produced a more stable thiosuccinimide conjugate in both rat and mouse sera. However, enzyme activity in mouse serum resulted in a loss of the PBD portion of the drug-linker in cleavable SG3544 and SG3249 ADCs. Despite linker cleavage in mouse serum, deconjugation via the retro-Michael reaction can still be monitored by mass spectrometry since the remaining linker can be observed and considered as a conjugated species. When cleaved linker is considered as a conjugated species, SG3544 ADC exhibited no retro-Michael deconjugation in mouse serum, whereas SG3249 ADC exhibited 38% retro-Michael deconjugation, indicating that a more stable thiol linkage resulted from the *N*-phenyl maleimide drug-linker. Enzymatic cleavage of drug-linker dipeptides in mouse serum at exposed mAb positions has recently been reported for drug-linkers containing val-cit, and therefore our data is not surprising considering that val-ala dipeptides are susceptible to the same enzyme [39]. Linker cleavage in mouse serum led to a loss of drug via a mechanism different than the retro-Michael reaction and probably impacted TGI studies, as discussed below.

ADCs that are prepared with non-cleavable PBD drug-linkers were not susceptible to mouse serum enzyme activity, and results were similar for samples that were incubated in rat or mouse serum. In both of the cases, a clear improvement in drug-retention for the ADC prepared with *N*-phenyl maleimide SG3683 was observed. For non-cleavable PBD drug-linkers the only mechanism for drug loss is the retro-Michael reaction, thus *N*-phenyl maleimide (and thiosuccinimide) functionality effectively reduced this process, resulting in more drug retention after seven days serum incubation. However, a small amount of drug loss was observed for ADC that was prepared with the *N*-phenyl maleimide SG3683 drug-linker, which could be due to the 20% unhydrolyzed thiosuccinimide species remaining in the isolated ADC product. However, *N*-phenyl thiosuccinimides are expected to hydrolyze quickly upon serum incubation at 37 °C (t_1/2_ = 1.5 h, pH 7.4, 37 °C) and retro-Michael mediated deconjugation is likely minimal. Another possibility for the apparent drug loss for the SG3683 ADC could be due to how mass spectrometry data was analyzed. The small shoulder of lower molecular weight (−44 amu) could be part of the intact ADC species with two fewer sodium counter ions. This peak was not included in data analysis and would lead to a higher drug content if included. Altogether, serum incubation results demonstrate that phenyl maleimide PBD drug-linkers could effectively reduce retro-Michael deconjugation at the thiol attachment point.

All of the PBD ADCs were potent cytotoxic agents in vitro, with low pM activities. Although drug loss from some ADCs was observed in serum stability assays, this did not impact the activity of ADCs in vitro, as indicated by the similar potencies that were observed between ADCs prepared with *N*-alkyl maleimide and *N*-phenyl maleimide drug-linkers. It is likely that ADC cell-binding and internalization occurs before drug-linker deconjugation by the retro-Michael process (T_1/2_ ~ 35–67 h) can impact results. Previously, we reported loss of ADC potency for unstable MMAE-conjugated ADCs in vitro, which was corrected by phenyl maleimide functionality in the drug-linker [12]. In that experiment, however, ADCs were incubated in serum prior to use in the cytotoxicity assay. In the current work, ADCs were added directly to cultured cells without pre-treatment. Altogether, in vitro results show that PBD ADCs prepared either *N*-alkyl or *N*-phenyl maleimide drug-linkers are selective and potent anticancer agents in vitro.

In vivo efficacy studies were conducted to determine if thiol conjugation through *N*-phenyl maleimide would provide a more durable response against tumors. Previous reports have demonstrated that stable thiol-linked ADCs perform better in vivo with MMAE drug-linkers, however, *N*-phenyl maleimide conjugates and PBD drug-linkers have not been evaluated [13]. ADCs that are prepared with cleavable drug-linkers showed similar TGI activity when dosed at 1 mg/kg, and a slight improvement was observed for *N*-phenyl maleimide SG3544 at 0.3 mg/kg. The benefit of stable thiol conjugation was not obvious for cleavable PBD drug-linkers and it is likely that dipeptide cleavage (as observed in mouse serum stability assays) affected ADC potency. Mouse serum stability results showed complete linker cleavage for both SG3249 and SG3544 ADCs after 7 days incubation. Although a detailed kinetic analysis was not performed, more linker cleavage (100%) than retro-Michael deconjugation (~40%) was observed after seven days incubation in mouse serum for the SG3249 ADC, suggesting that linker cleavage occurs faster than retro-Michael deconjugation. Thus, both SG3249 and SG3544 ADCs were likely inactivated by enzyme activity in mouse serum independent of the retro-Michael deconjugation process. We further evaluated the effect of linker cleavage in mouse serum by conjugating SG3249 and SG3544 to the known stable position resulting from the insertion of a cysteine at position 239 to yield A07-108-C239i ADCs [18]. This antibody position prevents drug-linker loss by both retro-Michael deconjugation and dipeptide cleavage mechanisms (Figure S3). A07-108-C239i ADCs conjugated with either SG3249 or SG3544 had significant TGI activity at both 1 mg/kg and 0.3 mg/kg (Figure S4). Thus, the susceptibility of val-ala linker cleavage in mouse serum at position T289C likely prevented the benefit of stable thiol conjugation to be realized. Enzymatic cleavage of dipeptide linkers in mouse serum for ADCs prepared at position T289C represents a limitation of this conjugation position to accurately evaluate ADC activity in mouse tumor models.

Non-cleavable PBD ADCs prepared by thiol conjugation through *N*-phenyl maleimide showed a clear improvement of in vivo anti-cancer activity when compared to the analogous *N*-alkyl maleimide construct. ADC prepared with the *N*-phenyl maleimide non-cleavable drug-linker SG3683 achieved tumor stasis at the 1 mg/kg dose, whereas ADC prepared with analogous *N*-alkyl maleimide drug-linker SG3376 showed no activity relative to controls at the same dose. When considering mouse serum stability assay results, ~40–50% PBD loss is expected after seven days for the SG3376 ADC construct. The loss of anti-tumor activity for the SG3376 ADC corroborates well with the observed drug loss in ex vivo mouse serum stability studies and supports the hypothesis that stable thiol conjugation of drug-linker through *N*-phenyl maleimide helps to maintain ADC potency under certain conditions.

Drug loss from the ADC may have more impact than simply lowering the drug content in the active agent. A distribution of species is likely produced by the deconjugation process, where some ADCs contain two drug-linkers, one drug-linker, or none at all. If the delivery of two drugs per mAb internalization event is required for effective cell killing, one-drug and no drug ADCs would serve as competitive inhibitors of ADCs with a higher drug load. This could be the reason why, although ~50–60% of drug is expected to be retained on the SG3376 ADC, an intermediate TGI effect was not observed. The exact rate of drug loss from ADCs in vivo could be determined by mass spectrometry-based PK studies, which we will consider in future work. Altogether, the lack of TGI activity observed with the unstable A07-108-T289C SG3376 ADC confirms the impact on conjugate stability on ADC performance in vivo, where, in this case, a *N*-phenyl maleimide-containing PBD drug-linker improved the in vivo activity of an ADC towards a solid tumor target.

## 5. Conclusions

*N*-phenyl maleimide stabilized the thiol-drug linkage in ADCs that were prepared by the conjugation of a PBD drug-linker to a solvent-exposed cysteine introduced at position T289C in the antibody framework. Spontaneous hydrolysis of the resulting *N*-phenyl thiosuccinimide conjugate resulted in the chemical stabilization of the drug-linker-antibody attachment point and also impacted the physical properties of the ADC, as evidenced by a reduced pI and an increased SYPRO Orange binding following thiosuccinimide hydrolysis. Despite chemical stabilization of the drug-linker attachment point, ADCs that were prepared with cleavable drug-linkers conjugated to position T289C were susceptible to mouse serum-specific cleavage of the dipeptide linker, which negated the in vivo benefit of a chemically stable thiol-drug-linker bond. Removal of the val-ala dipeptide in PBD drug-linkers eliminated the drug loss from the ADC that was caused by linker cleavage in mouse serum, thus isolating the retro-Michael reaction as the major mechanism for drug loss from the antibody. The combination of improved thiol-conjugate stability and a non-cleavable linker (i.e., SG3683) resulted in improved TGI activity in a mouse tumor model for an ADC prepared using a solvent-exposed position for conjugation, demonstrating that chemical stabilization of the drug-linker-antibody attachment point can overcome limitations in efficacy. Understanding the processes that affect ADC performance in vivo will allow for the evolution of drug-linkers that fine-tune properties and maximize therapeutic potential. The simple change to *N*-phenyl maleimide functionality in the PBD drug-linkers described here highlight the ability to precisely design ADC drug-linkers for desired properties that enable stable conjugation to antibody positions that are unstable for traditional *N*-alkyl maleimides.

## Figures and Tables

**Table 1:** Summary of antibody-drug conjugates (ADC) drug content and aggregate analysis.

ADC	DAR (rRP-HPLC)	DAR (MS)	Mass Added ^1^	Monomer (%)
A07-108-T289C-SG3544	1.8	1.6	1578.93 ^2^	97
A07-108-T289C-SG3249	1.7	1.6	1498.78	94
IgG-T289C SG3544 ^3^	1.7	1.8	1576.85 ^2^	95
A07-108-T289C-SG3683	1.7	1.7	1149.94 ^2^	93
A07-108-T289C-SG3376	1.7	1.8	1070.37	94
IgG-T289C-SG3683 ^3^	ND	1.8	1150.65 ^2^	95

^1^ Determined by mass spectrometry analysis of ADCs. Drug-linker molecular weights (g/mol) are; SG3249 = 1496.67, SG3544 = 1558.74, SG3376 = 1070.17, SG3683 = 1131.46. ^2^ Hydrolyzed thiosuccinimide species, +18 amu added to the expected mass. ^3^ IgG denotes a non-binding control antibody. Abbreviations: ADC, antibody drug conjugat; DAR, drug:antibody ratio; rRP-HPLC, reduced reverse phase high performance liquid chromatography; MS, mass spectrometry; ND, not determined.

**Table 2:** Summary ADC activities in vitro.

ADC	EC_50_ (ng/mL)	EC_50_ (pM)
A07-108-T289CSG3544	0.71 ± 0.12	4.7 ± 0.8
A07-108-T289C SG3249	1.5 ± 0.1	10 ± 0.7
IgG-T289C SG3544 ^1^	963 ± 150	6420 ± 1000
A07-108-T289C SG3683	0.45 ± 0.17	3.0 ± 1.1
A07-108-T289C SG3376	0.3 ± 0.18	2.0 ± 1.2
IgG-T289C SG3683 ^1^	25,959 ± 670	173,060 ± 4470

^1^ IgG denotes a non-binding control antibody. ADC, antibody-drug conjugates.

## Supplementary Materials

The following are available online at http://www.mdpi.com/2073-4468/6/4/20/s1. Figure S1: Representative SEC trace (IgG control T289C SG3376) showing peaks used to determine monomer content in ADCs, Figure S2: Identity and mass of species remaining conjugated to antibodies following linker cleavage in cleavable PBD drug-linkers, Figure S3: Mouse serum stability and tumor growth inhibition activity of A07-108 ADCs prepared with C239i cysteine engineered mAb.
