# Supplementary material for: Pyrrolobenzodiazepine Antibody-Drug Conjugates Designed for Stable Thiol Conjugation
Source: Antibodies (Basel). 2017 Nov 28;6(4):20. doi: 10.3390/antib6040020 (PMC6698857; doi:10.3390/antib6040020)
Supplement: Supplementary file 1 [file antibodies-06-00020-s001.pdf]

Supporting Information

Pyrrolobenzodiazepine Antibody-Drug Conjugates Designed for Stable Thiol Conjugation

R. James Christie[1], Arnaud C. Tiberghien[2], Qun Du[1], Binyam Bezabeh[1], Ryan Fleming[1], Amanda Shannon[1], Shenlan Mao[3], Shannon Breen[3], Jing Zhang[3], Haihong Zhong[3], Jay Harper[3], Herren Wu[1], Philip W. Howard[2], Changshou Gao[1]

1. Antibody Discovery and Protein Engineering, MedImmune LLC, Gaithersburg MD, USA
2. Oncology Research, MedImmune, Gaithersburg MD, USA
3. Spirogen Inc., London, UK

Table of Contents

1. Synthesis methods and characterization data for SG3544 and SG3683……………….……2-6
2. Mass spectra of ADCs showing an extended mass range………………………...……….7
3. Representative SEC trace showing aggregate determination…………………………...…...7
4. Linker cleavage products following mouse serum incubation…………………….……….8
5. Serum stability and tumor growth inhibition activity of A07-108 239 ADCs prepared by drug-linker conjugation at position 239…………………………….......…..9

Syntheses of SG3544 and SG3683

(a) Maleimide condensation, ring opening

Scheme:

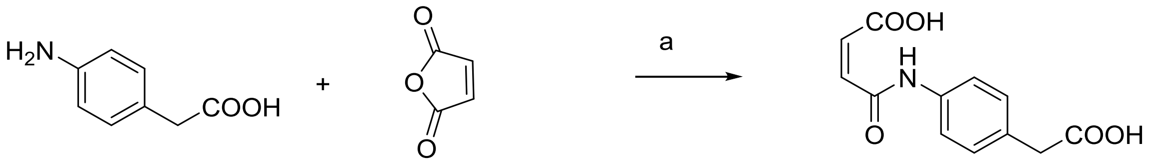

1 **2** **3**

Method: A suspension of 2-(4-aminophenyl)acetic acid (5 g, 33.1 mmol, 1 eq) in acetic acid (50 mL) was rapidly added to a cold solution (cold water bath, avoiding acetic acid freezing over) of maleic anhydride (3.26 g, 33.1 mmol, 1 eq) in acetic acid (25 mL). A rapid reaction followed, and a tan precipitate appeared. The reaction mixture was allowed to stir for 1h at room temperature and the precipitate was isolated by filtration and rinsed with water (50 mL). The solids were further purified by digestion in hot water (100 mL, 80°C), cooled, isolated by filtration and dried in a vacuum oven at 35°C overnight. Yield: 5.26 g, 64%.

Analytical Data: LC/MS, 0.99 min (ES+) m/z (relative intensity) 250.05 ($[M + H]^{+\cdot}$, 100); ^{1}H NMR (400 MHz, DMSO-d_6) δ 12.70 (s, 2H), 10.40 (s, 1H), 7.77 – 7.41 (m, 2H), 7.22 (d, *J* = 8.3 Hz, 2H), 6.47 (d, *J* = 12.1 Hz, 1H), 6.31 (d, *J* = 12.1 Hz, 1H), 3.53 (s, 2H).

Also see: Khan et. al., *J. Braz. Chem. Soc.*, Vol. 20, No. 2, 341-347, 2009

(b) Maleimide ring formation

Scheme:

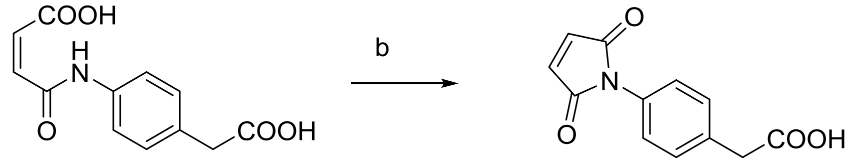

3 **4**

Method: Ring opened **3** (4.2 g, 16.8 mmol, 1eq) was refluxed overnight in acetic acid (60 mL). The acetic acid was rotoevaporated under vacuum and DCM (200 mL) was added to the residue to remove most of the coloured solid impurities. The solution was dried and the residue purified by chromatography (chloroform / MeOH 98/2 to 97/3). Fraction monitoring was done with EtOAc and potassium permanganate dip. Pale yellow solid. Yield 1.96 g (50%).

Analytical Data: LC/MS, 1.07 min (ES+) *m/z* (relative intensity) 231.90 ($[M+H]^{+}$, 100). ^{1}H NMR (400 MHz, Chloroform-*d*) δ 7.40 (d, J = 8.7 Hz, 2H), 7.33 (d, J = 8.6 Hz, 2H), 6.85 (s, 2H), 3.68 (s, 2H).

Also see: Sadownik and Philp, Angewandte Chemie, 2008, DOI: 10.1002/ange.200804223.

(c) Succinimide ester formation
Scheme:

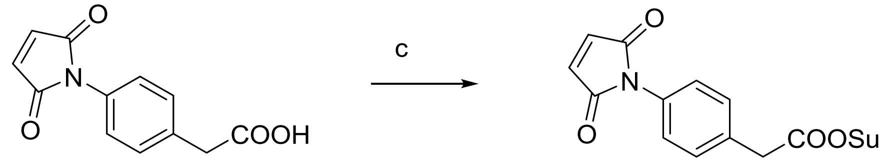

4 **5**

Method: Phenyl maleimide acid **4** (1 g, 4.32 mmol, 1 eq) was stirred in DCM with N-hydroxysuccinimide (498 mg, 4.32 mmol, 1 eq) and DCC (892 mg, 4.32 mmol, 1 eq). The reaction was monitored by TLC (EtOAc, $KMnO_4$ dip). Upon completion, the DCU was removed by filtration, the solution concentrated and purified by chromatography (75/25 EtOAc / Hexane, collection wavelength 223nM). Yield: 860 mg (61%).

Analytical Data: LC/MS, 1.24 min (ES+) *m/z* (relative intensity) 350.90 ($[M+Na]^+$, 100). ^1H NMR (400 MHz, Chloroform-d) δ 7.45 (d, J = 8.7 Hz, 2H), 7.38 (d, J = 8.6 Hz, 2H), 6.85 (s, 2H), 3.97 (s, 2H), 2.84 (s, 4H).

(d) PEG coupling
Scheme:

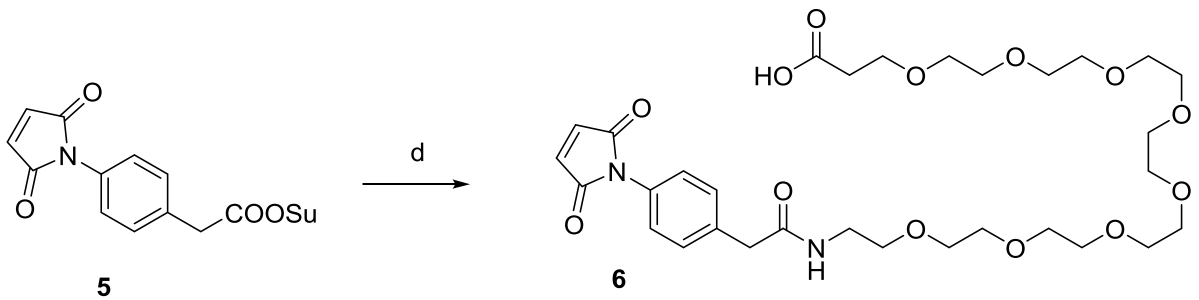

5 **6**

Method: Phenyl maleimide succinimide **5** (85 mg, 0.26 mmol, 1 eq) was stirred in DCM (5 mL) with Amino-$(Peg)_8$-Acid (114 mg, 0.26 mmol, 1 eq). The reaction was monitored by TLC (DCM/IPA: 80/20, $KMnO_4$ dip). Upon completion, the solution was concentrated and purified on a short silica column (DCM/IPA gradient from 95/5 up to 80/20; TLC monitoring with $KMnO_4$ dip on concentrated fractions). Yield: 61 mg (40%). The product was used immediately to minimise the chances of ring hydrolysis.

Analytical Data: LC/MS, 1.20 min (ES+) *m/z* (relative intensity) 655.45 ($[M+H]^+$, 100).

(e) SG3544
Scheme:

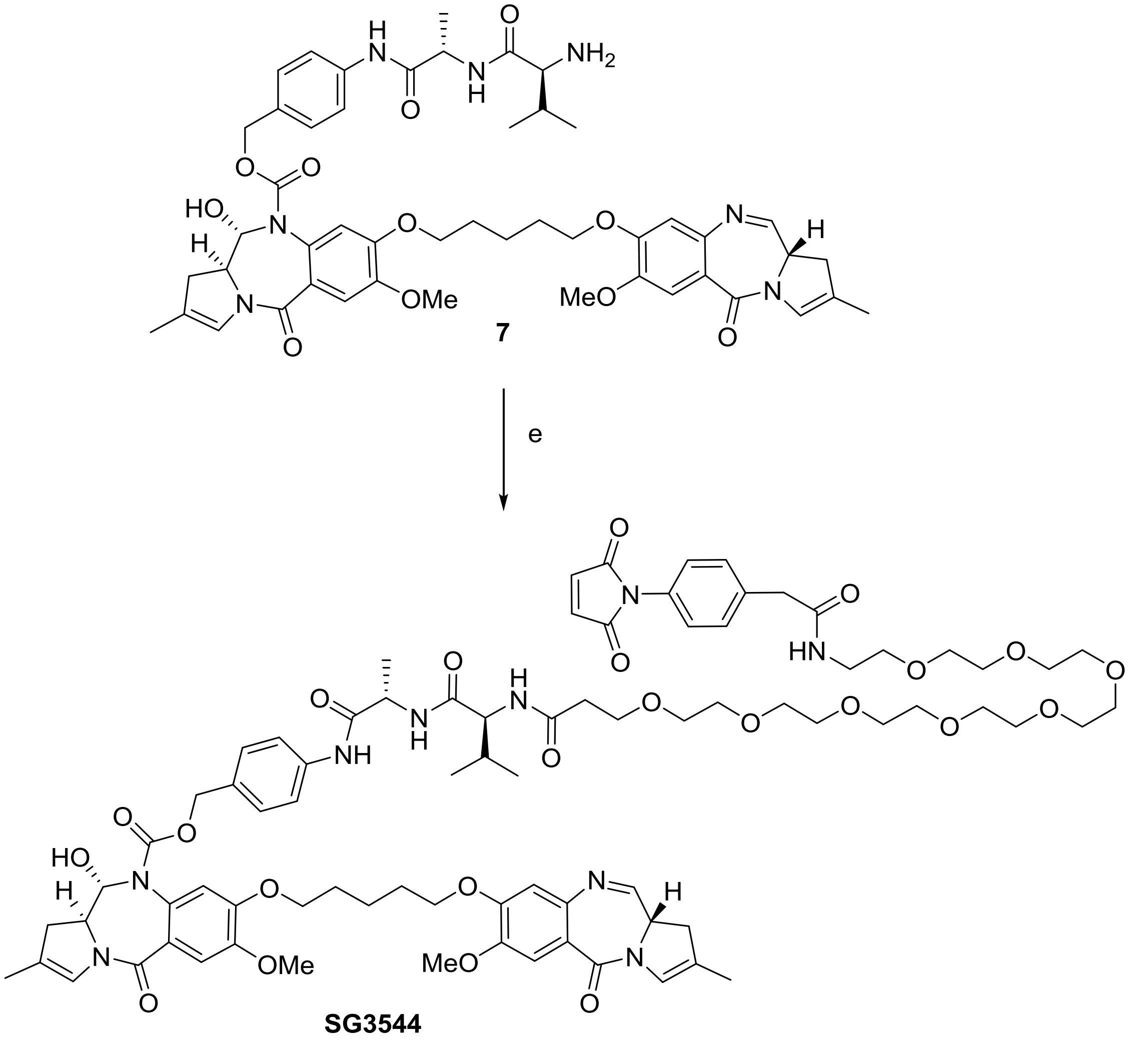

Method:
1-ethyl-3-(3'-dimethylaminopropyl)carbodiimide (25 mg, 0.129 mmol, 1 eq) was added to a solution of crude **7** (see Tiberghien et al., design and synthesis of tesirine, ACS med. Chem. lett., 2016) (119 mg, 0.129 mmol, 1eq) and Phenyl-Mal-(PEG)$_8$-acid **6** (85 mg, 0.129 mmol, 1 eq) in chloroform (5 mL). The reaction was stirred for 2 hours and the presence of starting material was no longer observed by LC/MS. The reaction mixture was concentrated and loaded directly on a silica gel column, followed by gradient elution (Chloroform/MeOH 98/2 up to 94/6). The purest fractions were collected and pooled to give 85 mg of material. Half of this material was purified by reverse phase preparative HPLC (0.01 % formic acid in acetonitrile and water, gradient) to give 13.84 mg of pure **SG3544**. In contrast the material which had not been purified by reverse

phase contained 8% of the ring-opened methyl ester (42.38 mg). The total yield was 28 %. A solution of free radical scavenger BHT in DCM (0.1% w/w BHT/**SG3544**) was added to the final aliquots as a stabilizer before drying.

Analytical Data: LC/MS, 1.50 min (ES+) *m/z* (relative intensity) 1559.10 ($[M+H]^{+\cdot}$, 20). ^1H NMR (400 MHz, DMSO-d6) δ 9.91 (s, 1H), 8.27 – 8.08 (m, 2H), 7.93 (d, J = 4.1 Hz, 1H), 7.86 (d, J = 8.5 Hz, 1H), 7.62 – 7.48 (m, 2H), 7.41 – 7.30 (m, 3H), 7.24 (d, J = 8.4 Hz, 2H), 7.17 (s, 4H), 7.05 (s, 1H), 6.84 (s, 1H), 6.74 (s, 1H), 6.64 (d, J = 10.9 Hz, 2H), 5.59 (s, 1H), 5.14 (s, 1H), 4.83 (s, 1H), 4.38 (s, 1H), 4.32 – 4.17 (m, 2H), 4.16 – 4.08 (m, 1H), 4.06 – 3.88 (m, 3H), 3.85 – 3.75 (m, 6H), 3.72 – 3.64 (m, 1H), 3.60 (s, 2H), 3.55 – 3.46 (m, 28H), 3.42 (t, J = 5.8 Hz, 2H), 3.28 – 3.17 (m, 2H), 3.12 – 2.83 (m, 4H), 2.47 – 2.27 (m, 4H), 1.96 (d, J = 6.6 Hz, 2H), 1.77 (d, J = 17.2 Hz, 10H), 1.56 (s, 2H), 1.33 – 1.20 (m, 3H), 0.85 (dd, J = 15.9, 6.7 Hz, 6H).

(f) SG3683
Scheme:

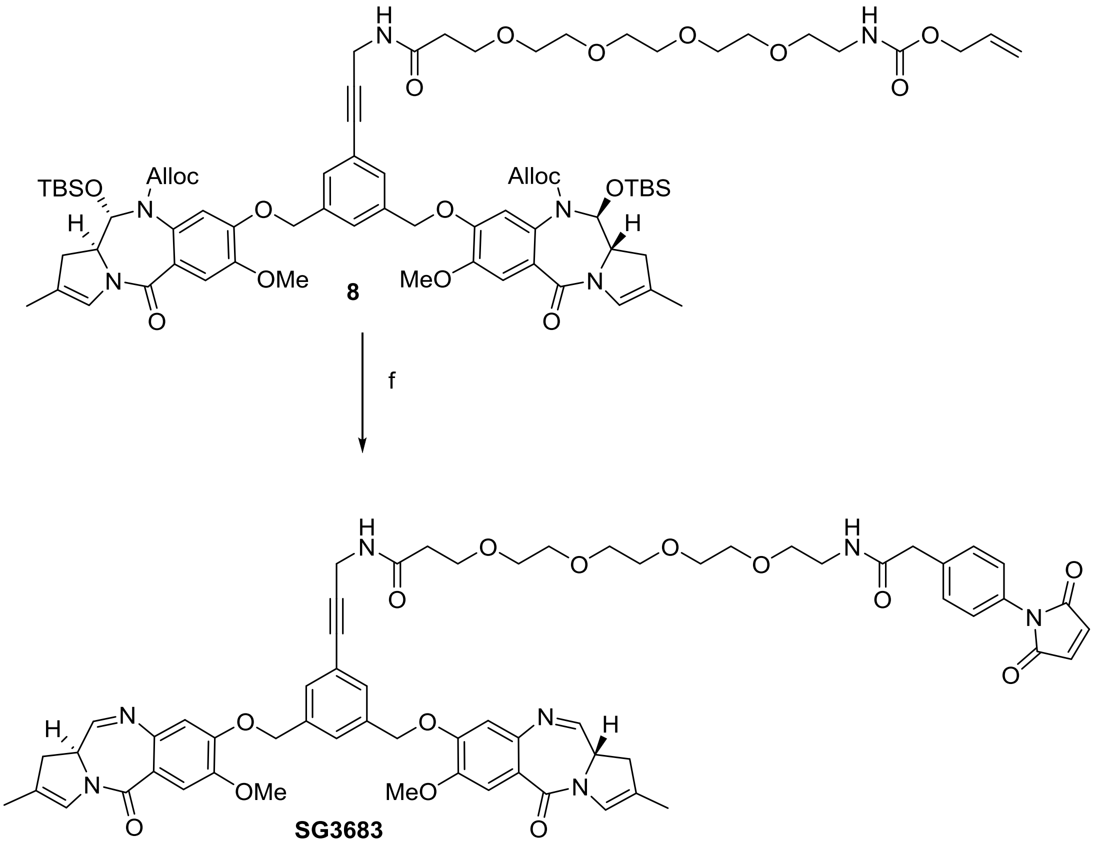

Method:
***Tetrakis*(triphenylphosphine)palladium(0)** (12 mg, 0.01 mmol, 0.04 eq) was added to a solution of **8** (see Tiberghien et al., An optimised synthesis of SG3376, tet. lett., 2017) (374 mg, 0.260 mmol, 1eq) and pyrrolidine (0.125 mL, 1.56 mmol, 6 eq) in dichloromethane (10 mL). The reaction was flushed with Argon once and stirred at room temperature for 2 hours. The reaction was diluted

with dichloromethane and stirred with saturated aqueous ammonium chloride. The organic phase was retrieved by passing the mixture through a phase separator, dried over magnesium sulphate, filtered, and excess dichloromethane removed by rotary evaporation under reduced pressure. The resulting crude amine (300 mg) was used directly in the next reaction.

1-ethyl-3-(3'-dimethylaminopropyl)carbodiimide (75 mg, 0.393 mmol, 1.5 eq) was added to a solution of crude amine (300 mg) and Phenyl-Mal-acid **4** (80 mg, 0.346 mmol, 1.3 eq) in DCM (5 mL). The reaction was stirred for 2 hours and the presence of starting material was no longer observed by LC/MS. The reaction mixture was concentrated and loaded directly on a isolera ultra 25g column, followed by gradient elution on Biotage Isolera (DCM/DCM +10% MeOH: 30/70 up to 0/100 in 12CV, elution from 60/40). The purest fractions (TLC 10% MeOH in DCM) were collected, pooled and rotoevaporated at 20˚C to give 139 mg of material (47%).

Analytical Data: LC/MS, 1.40 min (ES+) *m/z* (relative intensity) 566.95 ($[(M+2H)]^{2+\cdot}$, 100, 1133.25 ($[M+H]^{+\cdot}$, 20). ^{1}H NMR (400 MHz, Chloroform-d) δ 7.77 (d, J = 4.0 Hz, 2H), 7.52 (s, 2H), 7.48 – 7.40 (m, 3H), 7.40 – 7.35 (m, 2H), 7.32 – 7.27 (m, 2H), 6.92 (t, J = 5.3 Hz, 1H), 6.82 (s, 2H), 6.78 (s, 2H), 6.73 (p, J = 2.1 Hz, 2H), 6.48 – 6.39 (m, 1H), 5.13 (q, J = 12.6 Hz, 4H), 4.28 – 4.17 (m, 4H), 3.94 (s, 6H), 3.74 (t, J = 5.8 Hz, 2H), 3.67 – 3.45 (m, 16H), 3.45 – 3.35 (m, 2H), 3.16 (dd, J = 16.7, 11.6 Hz, 2H), 2.94 (dd, J = 16.6, 5.1 Hz, 2H), 2.49 (t, J = 5.8 Hz, 2H), 1.83 (q, J = 1.4 Hz, 6H).

General Experimental Conditions

Reaction progress was monitored by LCMS using the short run conditions outlined below. Extraction and chromatography solvents (HPLC grade) were bought from VWR, U.K. All other chemicals were purchased from Aldrich (standard reagent grades)

Column chromatography was performed on an Isolera (Biotage) automated system using normal phase SNAP Ultra cartridges purchased from Biotage.

The LC/MS conditions were as follows:

LCMS data were obtained using a Shimadzu Nexera series LC/MS with a Shimadzu LCMS-2020 quadrupole MS, with Electrospray ionisation. Mobile phase A - 0.1% formic acid in water. Mobile phase B - 0.1% formic acid in acetonitrile.

Gradient: initial composition was 5% B held over 0.25 min, then increase from 5% B to 100% B over a 2 min period. The composition was held for 0.50 min at 100% B, then returned to 5% B in 0.05 minutes and hold there for 0.05 min. Total gradient run time equals 3 min. Flow rate 0.8 mL/min. Wavelength detection range: 190 to 800 nm. Oven temperature: 50°C. Column: Waters Acquity UPLC BEH Shield RP18 1.7µm 2.1x50mm.

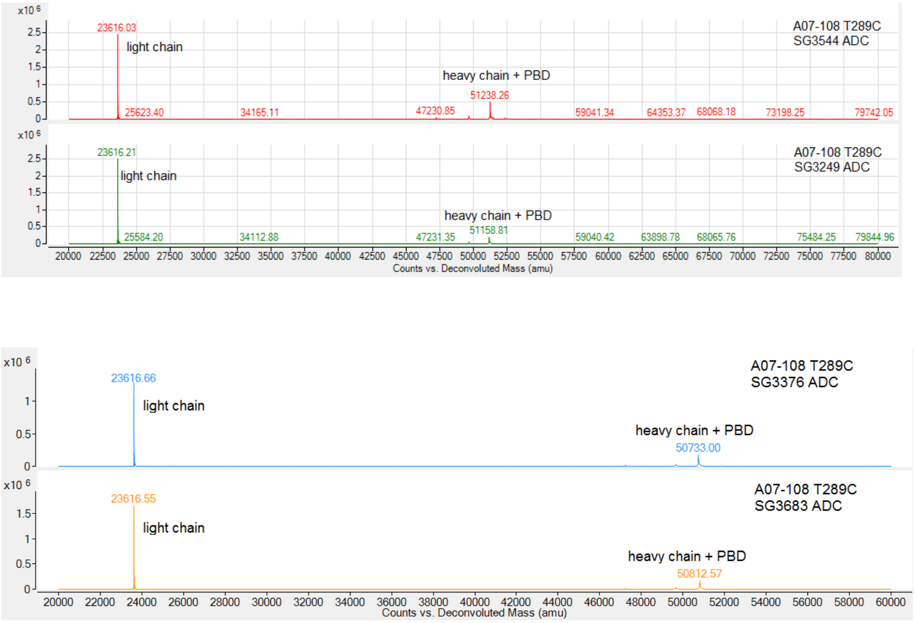

Figure S1. Reduced deglycosylated mass spectrometry analysis of ADCs showing an extended mass range.

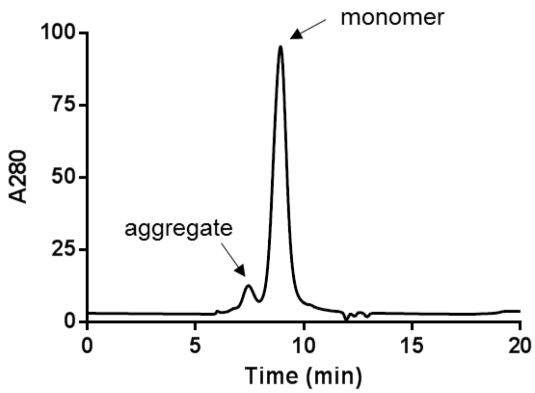

Figure S2. Representative SEC trace (IgG control T289C SG3376) showing peaks used to determine monomer content in ADCs.

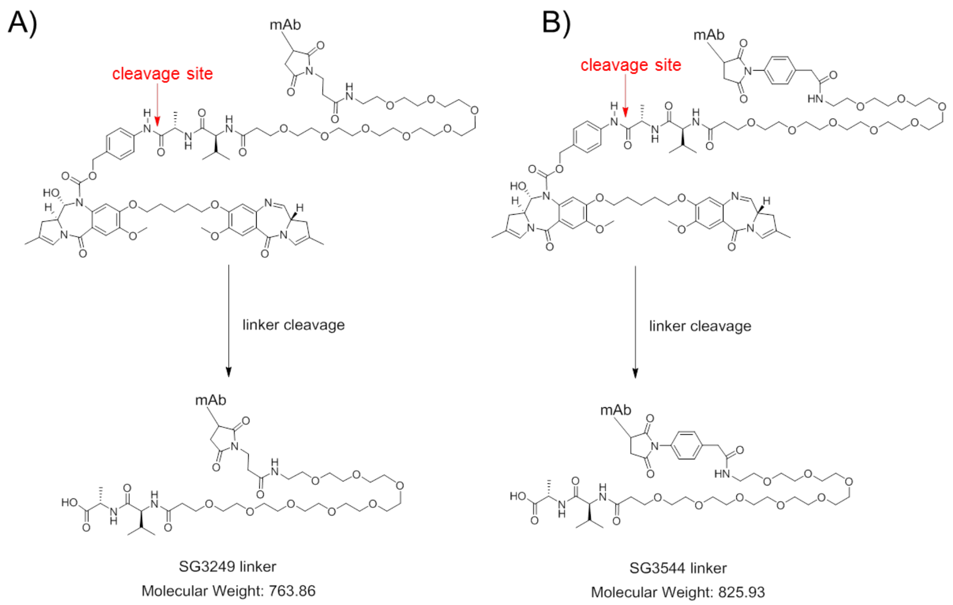

Figure S3. Identity and mass of species remaining conjugated to antibodies following linker cleavage in cleavable PBD drug-linkers. A) SG3249, B) SG3544.

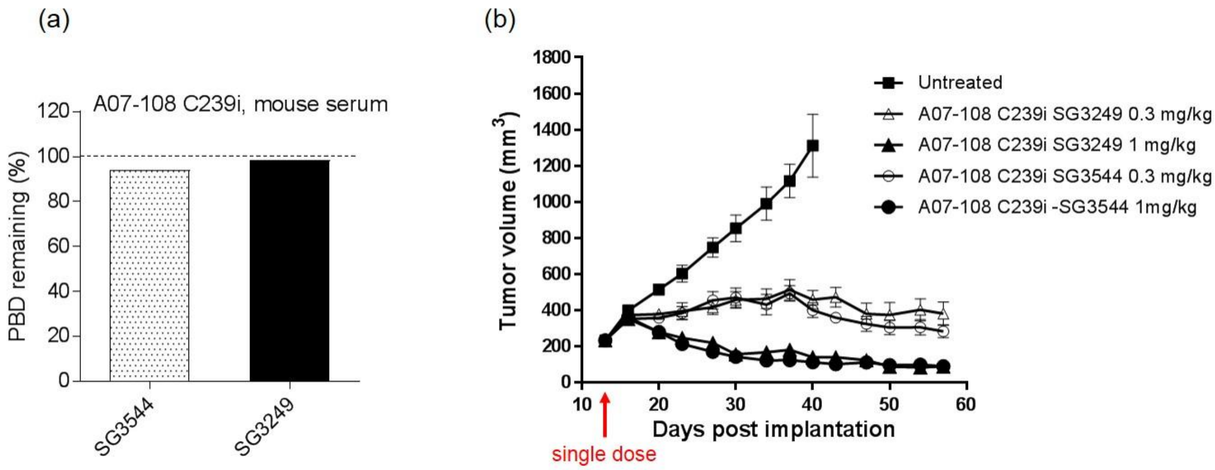

Figure S4. Mouse serum stability (a) and tumor growth inhibition activity (b) of A07-108 ADCs prepared with C239i cysteine engineered mAb. This position is not succeptible to retro-Michael mediated deconjugation and drug-linker cleavage in serum.
